# Supplementary material for: Role of Porcine Aminopeptidase N and Sialic Acids in Porcine Coronavirus Infections in Primary Porcine Enterocytes
Source: Viruses. 2020 Apr 5;12(4):402. doi: 10.3390/v12040402 (PMC7232180; doi:10.3390/v12040402)
Supplement: Supplementary file 1 [file viruses-12-00402-s001.pdf]

Supplementary figures

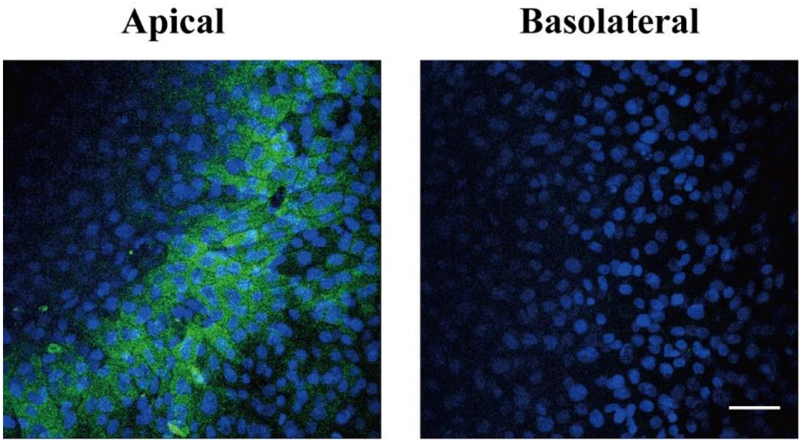

Figure 1. APN expression in the apical and the basolateral surfaces of enterocytes. Immunofluorescence staining of APN expression (green) in the apical and basolateral surfaces of enterocytes was performed 3 days post cultivation in the transwell system. Scale bar: 50μm.

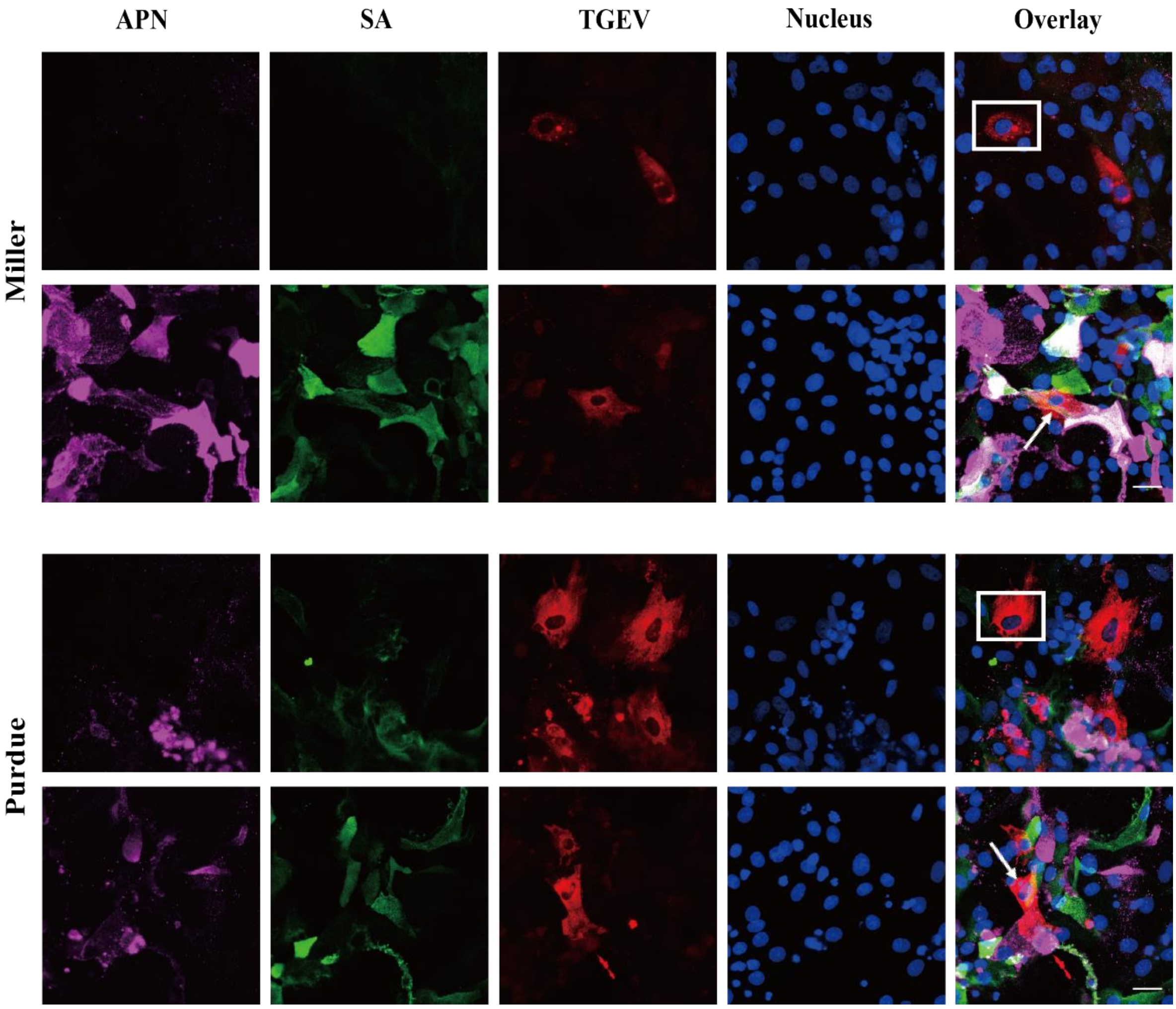

Figure 2. Immunofluorescence staining of TGEV infection in both APN and SA positive/negative enterocytes. Primary enterocytes were inoculated with TGEV for 24 h. Triple immunofluorescence staining was performed to visualize the TGEV infection in APN and SA positive/negative cells. APN

and SA double negative enterocytes that were infected with TGEV were indicated with a white square. APN and SA double positive enterocytes that were infected with TGEV were indicated with a white arrow. Scale bar: 25μm.
